# Vaccination uptake amongst older adults from minority ethnic backgrounds: A systematic review

**DOI:** 10.1371/journal.pmed.1003826

**Published:** 2021-11-04

**Authors:** Cini Bhanu, Dipesh P. Gopal, Kate Walters, Umar A. R. Chaudhry

**Affiliations:** 1 Research Department of Primary Care and Population Health, University College London, London, United Kingdom; 2 Institute of Population Health Sciences, Queen Mary University of London, London, United Kingdom; 3 Population Health Research Institute, St George’s, University of London, London, United Kingdom; Harvard University, Brigham and Women’s Hospital, UNITED STATES

## Abstract

**Background:**

Older adults from minority ethnic backgrounds are at increased risk of contracting COVID-19 and developing severe infection and have increased risk of mortality. Whilst an age-based vaccination approach prioritising older groups is being implemented worldwide, vaccine hesitancy is high amongst minority ethnic groups.

**Methods and findings:**

We conducted a systematic review and convergent synthesis to systematically examine perceptions of vaccinations amongst older adults from minority ethnic backgrounds. We included studies that reported on perceptions, beliefs, and attitudes towards vaccinations in older adults aged ≥65 years from a minority ethnic background. We excluded studies of vaccinations in investigation or development, studies focused on specific medical conditions, studies where ethnic background or age group was unidentifiable, systematic reviews, editorials, and conference abstracts. We searched MEDLINE, Embase, Virtual Health Library, Web of Science, Cochrane Library, medRxiv, and PROSPERO databases from inception to 15 July 2021. Risk of bias for studies was assessed using the Mixed Methods Appraisal Tool. The quality of evidence of collective outcomes was estimated using the Grading of Recommendations Assessment, Development and Evaluation–Confidence in the Evidence from Reviews of Qualitative research (GRADE–CERQual) framework. A total of 28 eligible studies conducted between 1997 and 2020 were included in the final analysis (17 quantitative surveys, 8 focus group or interview studies, 2 mixed methods studies, and 1 case–control study). The majority were US studies in English or Spanish, except for 6 studies set in Hong Kong, 2 studies in Japan, 1 study in Brazil, and 1 multi-centre study (including China, Indonesia, Turkey, South Korea, Greece, UK, Brazil, and Nigeria). In total, 28,262 individuals with an estimated mean age of 69.8 years were included, 63.2% of whom were female. We summarised the common concepts and themes across studies and populations using a convergent synthesis analysis. Thirteen themes categorised as barriers or facilitators were identified and grouped into structural factors—healthcare provider and system related, patient related, and policy and operational—and were analysed by minority ethnic group. The main limitation of the study was the predominance of studies from the US and East Asia.

**Conclusions:**

In this systematic review, we found that factors influencing vaccination uptake involve healthcare provider and system, patient-related, and governance-level factors that are specific to the older ethnic minority community being served. The evidence included in this review is supported by high or moderate certainty and can be translated to practice and policy. A tailored, multi-level approach combining increased education, access, and culturally competent discussions with trusted healthcare professionals to address health beliefs can maximise the potential impact of widespread vaccination policies.

## Abstract 2

In a systematic review, Dr. Cini Bhanu and colleagues investigate factors influencing vaccination uptake among older adults from minority ethnic backgrounds.

## Author summary

**Why was this study done?:**

- Older adults from minority ethnic backgrounds are at increased risk of contracting COVID-19 and developing severe infection and are at increased risk of mortality.
- Vaccine hesitancy is high amongst individuals from minority ethnic backgrounds, yet no studies to our knowledge have systematically reviewed the factors influencing vaccination uptake amongst older adults from minority ethnic backgrounds, who are high priority on current COVID-19 vaccination schedules.

**What did the researchers do and find?:**

- We conducted a systematic review identifying studies examining attitudes towards vaccination uptake amongst older adults (≥65 years) from minority ethnic backgrounds.
- We identified and summarised 13 themes categorised as barriers to and facilitators of vaccination uptake amongst older adults from minority ethnic backgrounds. These are related to healthcare provider and system factors, patient factors, and policy and operational factors.

**What do these findings mean?:**

- The factors influencing vaccination uptake amongst older adults from minority ethnic backgrounds identified in this review will help healthcare providers, clinicians, and policy makers tailor vaccination delivery to ensure adequate impact in this high-risk group, essential to the current COVID-19 pandemic and in future vaccination schedules.
- A tailored, multi-level approach combining increased education, access, and culturally competent discussions with trusted healthcare professionals to address health beliefs can maximise the potential impact, equity, and success of widespread vaccination policies.

## Introduction

Individuals from minority ethnic backgrounds are at increased risk of contracting coronavirus disease 2019 (COVID-19) and experiencing severe infection [1,2], and older adults are at highest absolute risk of COVID-19 mortality [3]. Whilst an age-based vaccination approach prioritising older groups is being implemented worldwide [4], vaccine hesitancy is high amongst ethnic minorities and in South Asian countries [5,6]. Lack of access to vaccines in low- and middle-income countries, coupled with vaccine hesitancy, could have significant implications for controlling the pandemic and for the global economic future [5,6]. Equitable vaccine distribution within high-income countries is also important, to prevent coronavirus mutation [7]. Achieving high vaccine uptake during the COVID-19 pandemic is a global priority.

The latest UK data show that 86% of people from White backgrounds aged 70–79 years have been vaccinated for COVID-19, compared to 55% of people from Black backgrounds [8]. Inequalities in delivery of healthcare reduce the effectiveness of health policies [9]. There have been studies investigating factors influencing uptake of childhood vaccinations amongst minority ethnic groups [10] and a systematic review by Nagata et al. in 2011 examining social determinants of influenza vaccination in older adults, which included some findings related to minority ethnic groups [11]. There are no studies to our knowledge specifically examining the views of older adults from minority ethnic groups towards vaccinations, of critical importance to the current COVID-19 pandemic. A recent study investigating predictors of COVID-19 vaccine hesitancy found that although older adults expressed a greater willingness to be vaccinated compared to their younger counterparts, individuals from Black, Pakistani, and Bangladeshi ethnic groups had greater reservations [12].

Lack of knowledge of the factors influencing vaccination uptake amongst high-risk older adults from minority ethnic groups limits the potential success of vaccination policies. Therefore, the aim of this review was to (1) examine perceptions of vaccinations amongst older adults from minority ethnic backgrounds, (2) summarise barriers to and facilitators of vaccination uptake, and (3) provide a resource to support vaccination uptake for use by clinicians and policy makers.

## Methods

### Search strategy and selection criteria

The search strategies were developed without language restrictions and included the databases of the Medical Literature Analysis and Retrieval System Online (MEDLINE; Ovid), the Excerpta Medica (Embase; Ovid), Virtual Health Library (VHL), Web of Science, Cochrane Library, medRxiv, and PROSPERO from inception to 15 July 2021. We used a search strategy combining terms and synonyms from referenced studies for ‘older adults’, ‘vaccinations’, ‘minority ethnic background’, and ‘views’ (S1 Text). We reviewed reference lists of eligible reports. This study is reported as per the Preferred Reporting Items for Systematic Reviews and Meta-Analyses (PRISMA) guideline (S2 Table). The study protocol is publicly available on PROSPERO (CRD42021237032).

Three independent reviewers systematically screened publications. Studies were eligible if they reported on perceptions, beliefs, and attitudes towards vaccinations in older adults from a minority ethnic background. We included studies from non-White majority populations in their country of origin as these are relevant to the experience of individuals from a minority ethnic background in other settings, since ethnic groups are considered to share a common ancestry, culture, and language [13]. ‘Older adults’ were defined as people aged ≥65 years, consistent with previous studies [11]. Studies of vaccinations in investigation or development, studies focused on specific medical conditions, studies where ethnic background or age group was unidentifiable, systematic reviews, editorials, and conference abstracts were excluded.

### Data screening

Records were downloaded into Endnote (version X9), and duplicates were removed. Using a standardised form, 3 independent reviewers (CB, DPG, and UARC) each conducted screening of two-thirds of the total number of titles and abstracts, and full-text articles. The Cohen’s κ statistic using the average across the 3 pairwise combinations of raters addressed inter-rater agreement regarding eligibility. Online systematic review software (Rayyan, QCRI) was used to facilitate literature screening. The publications were initially screened for title and abstract eligibility; full-text articles were then retrieved and screened for eligible publications to be included in data extraction. Discrepancies were resolved through discussion, if necessary with an adjudicator (KW).

### Data extraction and analysis

DPG and UARC independently extracted data from each article into a specified data extraction table. Data included study design, analysis method, geographical setting, language, sample size, mean age, ethnic background, sex, vaccination type, views towards vaccinations, and barriers and facilitators influencing vaccination uptake. A consensus meeting was held with CB to finalise data extraction. The risk of bias for studies was assessed by DPG and UARC using the Mixed Methods Appraisal Tool (MMAT), a validated tool for appraising methodological quality for use in systematic mixed study reviews [14]. A convergent synthesis approach informed by Pluye and Hong [15] was used to integrate qualitative and quantitative data. Results from studies that included qualitative, quantitative, and mixed methods data were transformed into qualitative findings using thematic synthesis, and a matrix was built on resultant themes and patterns agreed by CB, UARC, and DPG. Convergent qualitative synthesis seeks to address complex research questions to understand the ‘what, how, and why’ [15] relevant to the study question. Structural determinant categories identified in a previous study examining factors influencing vaccination uptake amongst general older adults [11] were drawn on to group themes. The quality of evidence of collective outcomes was estimated using the Grading of Recommendations Assessment, Development and Evaluation–Confidence in the Evidence from Reviews of Qualitative research (GRADE–CERQual) framework.

## Results

### Included studies

**Fig 1:**
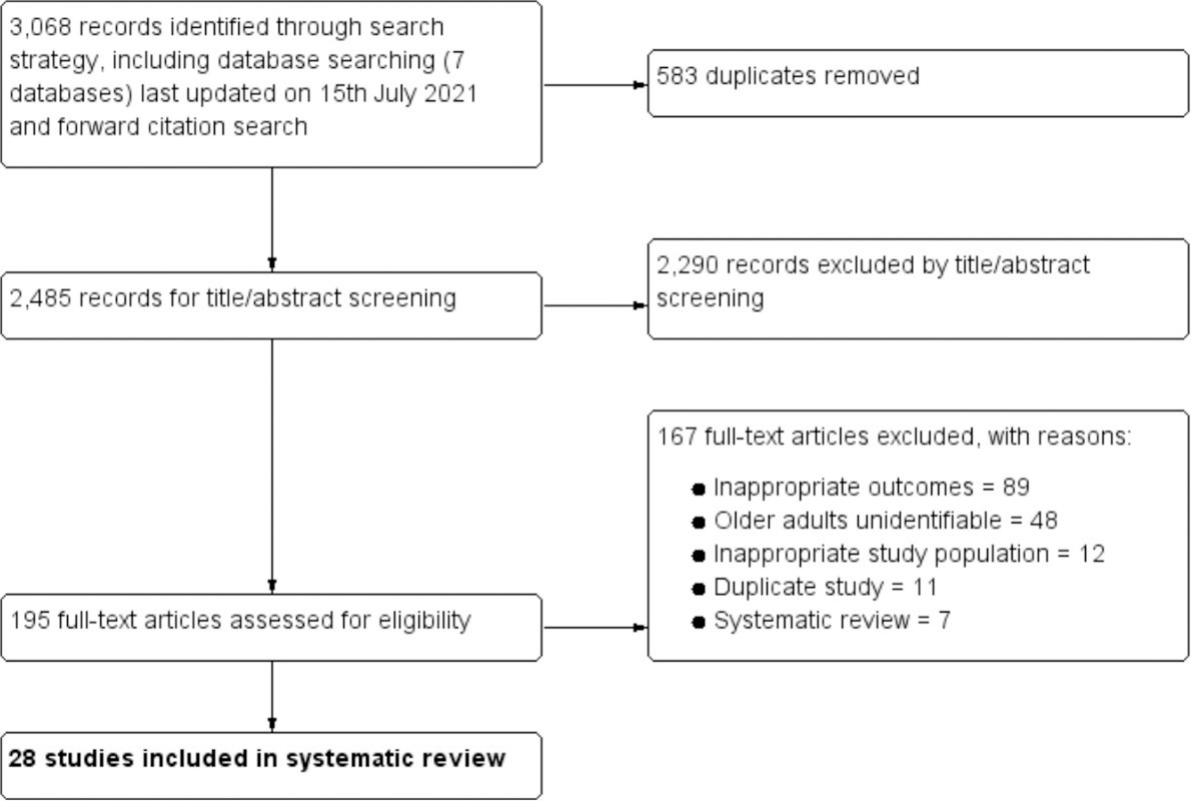
Preferred Reporting Items for Systematic Reviews and Meta-Analyses (PRISMA) flow diagram.

In total, 3,068 citations were identified by the search, 2,485 citations after duplicates were removed. Following title and abstract screening, 195 potentially eligible articles were retrieved in full text (Fig 1). Of the full-text articles, 167 were excluded: 89 did not examine the intended outcome, 48 were excluded because older adults were unidentifiable in a mixed age population, 12 were excluded because participants were either not from older age groups or not from a minority ethnic background, 7 studies were systematic reviews, and 11 studies were duplicates.

A total of 28 relevant studies conducted between 1997 and 2020 were included in the final analysis, of which 17 were quantitative surveys, 8 were focus group or interview studies, 2 were mixed methods studies, and 1 was a case–control study. There was substantial agreement between reviewers at the title and abstract stage (κ = 0.77) and full-text review stage (κ = 0.82) (reported as an average across 3 pairwise combinations of raters at each stage). Meta-analysis was not conducted since there was high heterogeneity across the studies in methods, reporting of outcome, and populations; few papers had sufficient quantitative data for meta-analysis; and this was not an a priori aim.

The MMAT risk of bias and GRADE–CERQual appraisals are summarised in S1 Fig and S1 Table, respectively. The evidence included is supported by high or moderate certainty.

### Study characteristics

Study characteristics of the 28 included studies are summarised in Table 1. The majority were US studies in English or Spanish, except for 6 studies set in Hong Kong, 2 studies in Japan, 1 study in Brazil, and 1 multi-centre study (including China, Indonesia, Turkey, South Korea, Europe, Greece, UK, Brazil, and Nigeria). In total, 28,262 individuals with an estimated mean age of 69.8 years were included, of whom 63.2% were female. Six studies explored views in participants from African American backgrounds, 2 studies in participants from Hispanic or Latinx backgrounds, 6 studies in participants from Hong Kong Chinese backgrounds, 2 studies in participants from Japanese backgrounds, 1 study in participants from Brazilian backgrounds, and 12 studies in mixed groups of older adults from minority ethnic backgrounds. Most studies explored views related to either the influenza or pneumococcal vaccine.

**Table 1:** Characteristics of studies.

Study	Year	Study setting (country)	Study design	Analysis	Number included	Estimated mean age	Language/cultural appropriateness	Ethnicities, as given by study	Percent female	Vaccine type
Abel [16]	2003	US	Survey	Chi^2^	1,006	75.2*	No mention but likely English	African American, Caucasian	61.4	Influenza
Albright [17]	2017	US	Focus groups	Team-based process; reflexive team analysis	68	52.1*	English or Spanish	English-speaking, Spanish-language	NS	Diphtheria, influenza, pertussis, pneumococcal, tetanus
CDC [18]	1997	US	Telephone survey	NS	600	NS	English or Spanish	Hispanic, White, other	NS	Influenza, pneumococcal, tetanus
Armstrong [19]	2001	US	Telephone survey	Chi^2^, log regression	486	76.6	No mention but likely English	African American, Caucasian, Hispanic	71.1	Influenza
Cameron [20]	2009	US	Focus groups	Extended parallel process model, latent content, and constant comparative techniques	48	74.1	No mention but likely English	African American	87.5	Influenza
Casarin [21]	2011	Brazil	Semi-structured interviews	Thematic analysis	7	69.4	Brazilian Portuguese	NS	57.1	Influenza
Chen [22]	2020	US	Telephone survey	Chi^2^, log regression	1,961	58.8	English or Spanish	Black, Filipino, Japanese, Latino, White	65	Influenza
Harris [23]	2006	US	Semi-structured in-depth interviews	Content analysis, including triangulation and constant comparison approach	20	71.5** (vaccinated); 74.0** (unvaccinated)	No mention but likely English	African American	70	Influenza, pneumococcal
Hebert [24]	2005	US	Survey	Logistic regression	6,746	74.5	No mention but likely English	African American, Hispanic, White	58.2	Influenza
Kajikawa [25]	2019	Japan	Questionnaire	Logistic regression	316	75**	Japanese	Japanese	48.4	Influenza
Kwong [26]	2008	Hong Kong, China	Survey	NS	70	75.8*	Chinese, likely Cantonese	Hong Kong Chinese	51.4	Influenza
Kwong [27]	2009	Hong Kong, China	Questionnaire	Chi^2^, *t* test, Kolmogorov–Smirnov test, logistic regression	197	75.2*	Chinese, likely Cantonese	Chinese	58.9	Influenza
Kwong [28]	2010	China, Indonesia, Turkey, South Korea, Greece, Canada, UK, Brazil, and Nigeria	Focus groups	Thematic analysis	208	75.5*	English and multiple non-English languages (not specified)	Multiple—Chinese, Indonesian, Turkish, Korean, Greek, Canadian, British	62.8	Influenza
Lasser [29]	2008	US	Survey and observation	Qualitative analysis	18	71.9	English or Spanish or Haitian Creole	Black, mixed, other, White	77.8	Influenza
Lau [30]	2008	Hong Kong, China	Telephone survey	Chi^2^, logistic regression	483	75.5*	NS	Hong Kong Chinese	55	Influenza
Nowalk [31]	2006	US	Telephone survey	Chi^2^, logistic regression	375	64.9*	English	African American	63	Influenza, pneumococcal
Phippard [32]	2013	US	Survey	Chi^2^, logistic regression	197	46.5*	English or Spanish (culturally appropriate)	Mexican	70.1	Influenza
Ramanadhan [33]	2015	US	Online survey	ANOVA, Chi^2^	1,569	44*	English	African American, Hispanic	56	Influenza
Rikin [34]	2017	US	Survey	Wilcoxon, logistic regression	200	74	English or Spanish (culturally appropriate, with translators)	Hispanic	73	Influenza
Schwartz [35]	2006	US	Survey	Chi^2^, logistic regression	454	77.5*	English	African American	54.4	Influenza
Sengupta [36]	2004	US	Interviews	Thematic coding	28	74.9	English	African American	78.6	Influenza
Singleton [37]	2005	US	Survey	Chi^2^, log regression	1,839	77.6*	English	Black, Hispanic	58.6	Influenza, pneumococcal
Siu [38]	2021	Hong Kong, China	Interviews	Thematic coding	40	72.7	Cantonese	Hong Kong Chinese	67.5	Influenza, pneumococcal
Sun [39]	2020	Hong Kong, China	Survey and focus groups	Chi^2^, log regression	2,452	51.4*	Cantonese	Hong Kong Chinese	64.8	Influenza
Takahashi [40]	2003	Japan	Case–control	Chi^2^, *t* test, Mann–Whitney, logistic regression	210	69.8	No mention but likely Japanese	Japanese	66.7	Influenza
Winston [41]	2006	US	Telephone survey	Chi^2^, multivariate binomial regression	4,577	74.5	No mention but likely English	Black, Hispanic, White	Unknown; ‘majority’ stated	Influenza, pneumococcal
Wooten [42]	2012	US	Telephone survey	Chi^2^, logistic regression	3,821	77.8*	No mention but likely English	Black, Hispanic, other, White	64.7	Influenza, pneumococcal
Yu [43]	2014	Hong Kong, China	Questionnaire	Logistic regression	306	74.6	Chinese, likely Cantonese	Chinese	37.9	Influenza

*Mean age estimated using sum total of each subgroup frequency multiplied by mid-point of each range divided by total frequency.

**Median age used.

ANOVA, analysis of variance; CDC, Centers for Disease Control and Prevention; NS, not stated.

### Synthesis

**Fig 2:**
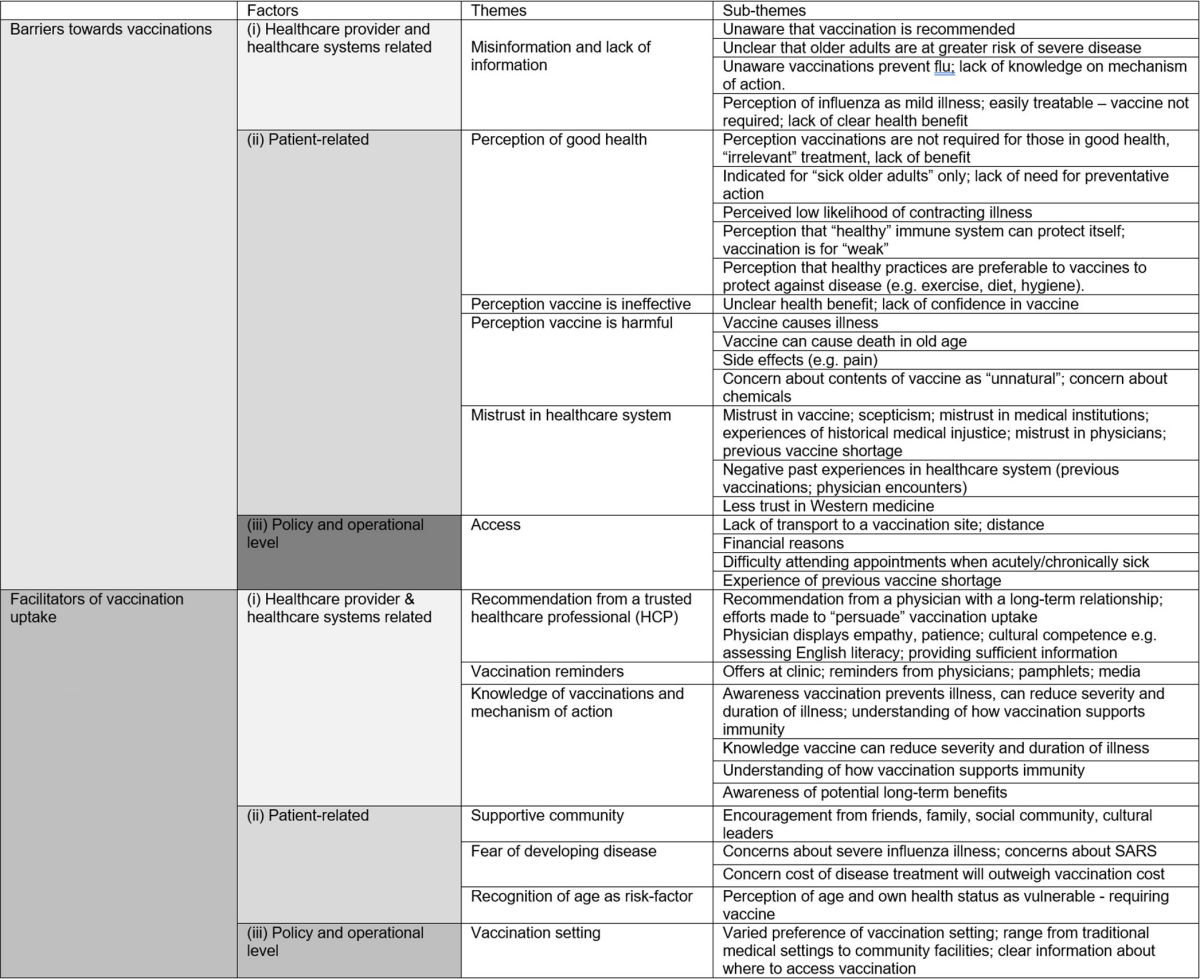
Barriers to and facilitators of vaccination uptake amongst older adults from minority ethnic backgrounds. SARS, severe acute respiratory syndrome.

The following sections outline the themes that were identified as barriers to and facilitators of vaccination uptake amongst older adults from minority ethnic backgrounds. Themes were grouped according to previously established structural determinants: (1) healthcare provider and system related, (2) patient related, and (3) policy and operational [11] (Fig 2).

### Barriers to vaccination uptake

#### Misinformation and lack of information on vaccines

A significant lack of information around the need for vaccination, potential benefit, and mechanisms underpinning how vaccinations work was identified as an important theme across the studies and populations. Older adults from African American, Hispanic, Nigerian, and Indonesian backgrounds were more likely to report they were uninformed or unaware that the influenza vaccination was recommended for them [17,20,24,26,28,34,37]. In a US study, individuals from Hispanic backgrounds were 12.6% more likely than White individuals to be unaware that the flu vaccination was needed [24].

Common barriers amongst African American communities were a lack of awareness of how vaccinations could prevent disease or benefit health, the perception that influenza is a mild illness that does not require preventative measures [20], a perceived low likelihood of contracting influenza [37], and a lack of awareness that older adults are at greater risk of severe illness [20]. Similar views were shared amongst individuals from Western Pacific backgrounds; influenza was considered ‘easily treatable’ [39], and only a third of older adults from Hong Kong Chinese backgrounds felt they were susceptible to influenza or perceived it as a serious illness [26].

#### Perception of good health

The perception of being in good health, and therefore not requiring a vaccine, acted as a common barrier. Older adults from African American communities perceived vaccinations as being irrelevant to ‘healthy’ people and as being indicated for older adults who were sick or had chronic disease [20,23,36]. Many associated healthcare use with illness, rather than preventative care.

Similar perceptions were expressed by individuals from Hong Kong Chinese backgrounds, who considered a healthy body an indication of a strong immune system that can protect itself [26,28,39]. Siu et al. described views that vaccines are for the ‘weak’ (particularly amongst older men), that experiencing viral illness can strengthen the body, and that a healthy body with a healthy ‘root’ does not require vaccination, reflective of traditional Chinese medicine principles [38]. Participants from Turkey, Canada, and the UK shared views that a healthy lifestyle—including sufficient exercise, a balanced diet, and good hygiene practices—was preferable to protect against influenza [28].

#### Perception vaccine is ineffective

Older adults across the majority of studies and populations believed vaccinations were unlikely to be effective at preventing disease [18,20,26,30,35,39].

#### Perception vaccine causes harm

A strong deterrent identified amongst African American older adults was the belief that vaccinations cause illness, particularly that they cause the flu [16,20,22,23,35,41]. Approximately 32% of unvaccinated African Americans believed that influenza vaccination causes influenza, compared to 18% of White Americans [22]. A further study reported that African Americans were 10.8% more likely to believe that flu vaccination causes the flu [16]. This perception was mirrored in studies exploring the views of multiple populations including individuals from African American, Hispanic, and Western Pacific backgrounds [24,26,28,29,34,43] and older adults from Brazilian backgrounds [21]. Older adults from Brazilian backgrounds were concerned the vaccination may even cause death in old age [21].

Older adults from African American, Hispanic, Brazilian, and Western Pacific backgrounds also cited side effects of vaccination as a major concern [20,21,24–27,34,35,37,39,43]. Fear of pain, allergic reactions, and generalised symptoms that would interfere with daily life was reported.

Some older adults from Hispanic backgrounds believed vaccines could harm the immune system and would prefer alternative medicines [34].

#### Mistrust in the healthcare system

Scepticism of vaccines and mistrust in physicians and the healthcare system were cited frequently by older adults from African American backgrounds [20,22,23,33,36]. Harris et al. acknowledged that mistrust in medical institutions was a product of historical abuses experienced by African Americans, historical medical injustice, and prior negative experiences with healthcare [23].

Ramanadhan et al. described mistrust in vaccines amongst older adults from Hispanic backgrounds, but found they were more likely to be open to persuasion if given further information, compared to older people from African American backgrounds [33].

Mistrust amongst older adults from Hong Kong Chinese and Japanese backgrounds centred around a scepticism of Western medicine [38,40]. The studies illustrate perceptions of vaccinations as ‘unnatural’, ‘chemical’, and ‘strong’ compared to traditional Chinese medicine.

#### Access

A significant theme across older Hispanic communities was issues related to vaccination access, including lack of transport, cost, distance to vaccination centres, and concern about travelling if unwell [18,22,29]. This appeared to be a greater concern compared to other issues, such as mistrust in vaccines, in this community [18]. Approximately 13% of older adults from Latinx backgrounds cited access and cost issues as the main reason for non-vaccination, compared to 2% for individuals from other racial/ethnic groups [22].

Transport and cost were also cited as barriers amongst individuals from African American communities [36], Western Pacific backgrounds [30,40], and the multi-centre study [28]. Kwong et al. highlighted differences in vaccine affordability and availability due to different healthcare finance systems in their multi-centre study [29]. In Turkey, China, and Nigeria, where vaccination is paid for by the individual, affordability is a predominant barrier [28]. Availability was a key barrier in Brazil, where the health system relies on the private sector, and vaccine shortage was a key barrier in Greece [28].

### Facilitators of vaccination uptake

#### Recommendation from a trusted healthcare professional

A strong theme across many studies was the positive effect of receiving a recommendation or advice about vaccinations from a trusted healthcare professional (HCP). Older adults from African American backgrounds were less likely to have reservations about vaccinations if they were recommended by a physician [16,20,35,41]. Similar views were shared in studies including older adults from mixed minority ethnic backgrounds [18,29,42], Japanese backgrounds [25], and Chinese backgrounds [27]. Lasser et al. highlighted the importance of a trusting compassionate relationship with the HCP, cultural competence (e.g., a physician taking the time to assess English literacy before providing vaccination information), empathy (‘treating the patient as a person’), and the ability to adapt to individual needs [29].

#### Vaccination reminders

Vaccination reminders were a facilitator of vaccination uptake amongst older adults from African American backgrounds [31,36], Hispanic backgrounds [17,18], Hong Kong Chinese backgrounds [39], and Turkish and South Korean backgrounds [28]. Reminders from a physician were most frequently cited as positive factors (some felt this displayed care from their HCP, increasing trust in the relationship) [17], followed by offers from a clinic, posters, pamphlets, and reminders in the media.

#### Supportive community

A consistent theme across most studies was the importance of positive views of vaccinations and encouragement from an older person’s social community (including family, friends, and cultural and religious leaders). Older adults from African American backgrounds [31,36], Hispanic backgrounds [18,34], Greek backgrounds [28], and Western Pacific backgrounds [27,28,39,40] shared this perspective. Close social contacts and community were perceived as trusted sources. Conversely, negative opinions amongst the older persons’ social network acted as a barrier to uptake [34,36].

#### Fear of developing disease

A fear of developing infectious disease acted as a facilitator of vaccination uptake amongst older adults from Western Pacific backgrounds [26–28,30,40,43]—with fear of severe acute respiratory syndrome (SARS) cited in 1 study [30]. Chen et al. found this perception was consistent across populations of African American, Hispanic, Japanese, and Filipino backgrounds and similar across age ranges amongst older adults [22]. Chen et al. also reported that a significantly greater proportion of Japanese Americans (27%) and Filipino Americans (37%) were very concerned about getting influenza compared to older adults from White backgrounds (20%) (*p* < 0.01) [22]. Older adults from Chinese backgrounds believed the cost of developing the disease would far outweigh the cost of vaccination, as a facilitator [28].

#### Knowledge of vaccinations and their mechanism of action

Sufficient information about how vaccinations prevent disease, and awareness that vaccinations are able to reduce the severity and duration of illness, were a positive influencer amongst older adults from African American backgrounds [20,23,36] and Western Pacific backgrounds [25,26,40]. Older adults from mixed minority ethnic backgrounds in the study by Lasser et al. reported that having evidence that a vaccine had long-term effects lasting for more than 6 months was a positive influencer [29].

#### Recognition of age as a risk factor

The perception of older age as a risk factor for severe infection was a positive influencer of vaccine uptake amongst older adults from African American backgrounds [23,36], Hong Kong Chinese backgrounds [26,43], Japanese backgrounds [25], and Mexican backgrounds [32]. Sengupta et al. identified views that the perception of one’s health as vulnerable and having multiple comorbidities related to older age also acted as facilitators amongst individuals from African American backgrounds [36].

#### Vaccination setting

Preference of vaccination setting varied across the populations examined in the studies. Older people from African American backgrounds expressed greater trust in traditional medical settings (such as a clinic or hospital) compared to community centres [16]. The majority of older people from Hispanic backgrounds in the study by the Centers for Disease Control and Prevention preferred a busier community-located setting [18]. For older adults from Hong Kong Chinese backgrounds, clear information about where vaccination centres were located was most important [30].

## Discussion

This systematic review has, to our knowledge for the first time, summarised existing evidence on factors influencing vaccination uptake amongst older adults from minority ethnic backgrounds, which is of high relevance to the current COVID-19 pandemic. It presents essential healthcare-provider-related, patient-related, and policy-related factors to consider in vaccination strategies currently being rolled out to ensure adequate impact, efficacy, and equity. These findings are based on high- to moderate-certainty evidence, which can be translated to practice and policy.

Lack of information about how vaccinations prevent illness, and misconceptions around efficacy, side effects, and perceived low risk of infectious disease, were fundamental barriers to vaccine uptake amongst older adults from all minority ethnic backgrounds included in this study. Views that vaccinations are irrelevant to healthy older people and are indicated for those with poorer health status were shared amongst individuals from African American and Western Pacific backgrounds and minority ethnic groups in Turkey, Canada, and the UK. Access and cost were large negative influencers amongst people from Hispanic, Greek, Nigerian, and Turkish backgrounds; historic distrust of healthcare establishments was important amongst African American communities; and the belief that vaccination conflicts with traditional Chinese medicine was significant amongst those from Western Pacific backgrounds. Facilitators were common amongst older adults across all minority ethnic backgrounds included in the studies. Adequate knowledge of how vaccines achieve health benefits, recognising age as a risk factor for serious illness, fear of developing disease, advice from a trusted HCP, reminders, and encouragement from an older person’s social community were positive influencers of vaccine uptake.

### Comparison to other literature

We have grouped themes under important structural determinants identified by Nagata et al. [11]. Similar sub-themes relevant to older adults from minority ethnic backgrounds emerged in our study: fear and mistrust of modern medicine amongst older adults from African American backgrounds, language and literacy barriers, and cultural beliefs that natural, healthy lifestyles are preferable to vaccinations [11].

A systematic review by Bish et al. [44] examining factors associated with influenza vaccination uptake in the general population found that the degree of fear related to the 2009 influenza pandemic outbreak, positive social pressure, and less fear of side effects correlated with increased intentions of vaccination uptake, consistent with our findings. This suggests these factors are common to the general population. Other factors identified in our study—including lack of information on vaccines, the belief that vaccines cause disease, the perception that vaccines are irrelevant when in good health, and access issues as barriers to vaccination uptake and fear of the disease as a facilitator of vaccination uptake—are likely more common in older adults from minority ethnic backgrounds and important to consider in COVID-19 age-based vaccination policies.

Sheldenkar et al. [45] recently conducted a systematic review of general adult influenza vaccine acceptance in Asia. Similar to our findings, there was a significant lack of studies in South Asia compared to East Asian countries (with Hong Kong having the greatest number of publications). This highlights a pressing gap in the research. South Asians are a sizable ethnic minority in many European countries [46], previous studies have identified vaccine hesitancy amongst Pakistani and Bangladeshi ethnic groups [12], and these groups are at increased risk of COVID-19 mortality [4].

### Strengths and limitations

This is the first systematic review to our knowledge seeking to understand perceptions and beliefs influencing vaccination uptake amongst older adults from minority ethnic backgrounds. It provides insights that are pertinent to the COVID-19 pandemic, with vaccination policies being rolled out worldwide. The findings also inform existing national vaccination schedules and future policies in epidemics, to ensure optimal impact and equity. A further key strength of this study is the focus on older adults. Despite being the most vulnerable to severe illness from communicable disease [3], older adults are often excluded from clinical research [47]. The main limitation is the limited number and range of studies available, with the vast majority being set in the US, the findings of which may not be applicable to ethnic minority groups in other settings. We included a handful of studies from majority populations in East Asia, which may not represent the views of those groups in settings where they are an ethnic minority, but we felt these studies were still likely to be of relevance to these populations. There were insufficient papers across diverse countries and ethnicities to examine cross-country differences in majority populations.

Many studies were excluded as the perspectives of older participants were unidentifiable in a mixed age population. However, a brief overview of these studies indicated that the main themes were broadly similar, though views may be more diverse amongst younger populations.

All studies identified in this review were on influenza and pneumococcal vaccinations. Whilst these vaccinations are largely comparable to COVID-19 vaccination, some recommendations may not be directly applicable to the COVID-19 context. We identified some studies related to COVID-19 vaccination and minority ethnic groups in our search; however, these were all excluded due to age criteria or their focus on vaccinations in development. Recent studies that have explored COVID-19 vaccination hesitancy amongst minority ethnic groups of all ages have reported similar barriers to those we have identified: perceived risk of getting infected with COVID-19, concerns about side effects and safety, and medical mistrust amongst Hispanic and African Americans [48,49].

The studies included in our systematic review focused on minority ethnic populations, but smaller intra-group differences may have been obscured. Ethnic groups are considered to share a common ancestry, culture, and feeling of solidarity with one another [13]. There is wide variation within minority ethnic groups in country of origin, language, religion, socioeconomic characteristics, and experiences that limit the interpretations we can make from this study, but enough shared culture with regards to family structures, identity, and health beliefs to make ethnic identity relevant to health behaviours [13]. Our outcome focused on the perceptions of and attitudes towards vaccinations amongst minority ethnic groups. We acknowledge that whilst the themes cover some social determinants that influence vaccination uptake, all potential social determinants that pose barriers to vaccination in minority populations (including broader economic, social, and cultural factors) may not have emerged in the data.

The majority of studies did not report on the immigration status or citizenship of the individuals in the minority ethnic groups, which is a further limitation. In our search, we identified 2 studies examining perceptions of vaccinations in migrant groups [50,51]; however, these were excluded due to age criteria. Whilst some of the barriers in migrant groups reported in these studies mirrored those identified in this review (fear of side effects, mistrust in the healthcare system, and access), others were unique to this population (fear of facing immigration checks, lack of information in an appropriate language, and incompatibility with migrants’ religion) [50,51]. Language barriers were not identified as an emergent theme in our review. The majority of non-US studies were conducted in either the native language of the minority ethnic group and/or indicated that a translator was used. Some US studies covering Hispanic populations were conducted in Spanish or used a culturally appropriate translator; however, 11 US studies including African American and Hispanic participants either stated the study language was English or language was not specified, which is an important limitation. Clear reporting on language and use of appropriate methods is recommended for future studies.

### Recommendations for practice

This review recommends, on a broad level, that efforts to provide adequate information and dispel misconceptions around vaccines by healthcare providers are fundamental to facilitating vaccine acceptance. Older adults from minority ethnic backgrounds who perceive themselves as healthy may benefit most from targeted intervention to increase uptake. Intervention is likely to be most effective through long-term trusted relationships with HCPs, tailored conversations (including family and friends), and a compassionate exploration of patient-related health beliefs. This can be supported by healthcare-system-level actions such as vaccination reminders and translated written information. Policy- and governance-level actions should focus on increasing access. This should include addressing transport issues, providing access for older adults living in rural communities, ensuring adequate availability of vaccination centres, providing home-based vaccination for frail older adults, incorporating vaccination sites into community facilities, and addressing financial barriers and existing inequity in access to preventative healthcare. Healthcare providers and policy makers should seek to tailor these recommendations to the needs and patient-related factors specific to the older ethnic minority communities they serve. Future research should address the gap in studies seeking to understand attitudes to vaccinations amongst older adults from ethnic minority groups in countries outside the US, including Europe and Australasia, and in majority groups in South Asia and Africa.

## Conclusion

Moderate- to high-quality evidence shows that factors influencing vaccination uptake by older adults from minority ethnic backgrounds involve healthcare provider and system factors, patient-related factors, and governance-level factors that are specific to the older ethnic minority communities being served. A tailored, multi-level approach combining increased education, increased access, and culturally competent discussions with trusted HCPs to address health beliefs can maximise the potential impact of widespread vaccination policies.

## Supporting information

S1 Fig MMAT appraisals. (DOCX)

S1 Table GRADE–CERQual assessment. (DOCX)

S2 Table PRISMA checklist. (DOCX)

S1 Text Search strategy for MEDLINE. (DOCX)

## Abbreviations

COVID-19: coronavirus disease 2019
GRADE–CERQual: Grading of Recommendations Assessment, Development and Evaluation–Confidence in the Evidence from Reviews of Qualitative research
HCP: healthcare professional
MMAT: Mixed Methods Appraisal Tool
