# Supplementary material for: Vaccination uptake amongst older adults from minority ethnic backgrounds: A systematic review
Source: PLoS Med. 2021 Nov 4;18(11):e1003826. doi: 10.1371/journal.pmed.1003826 (PMC8568150; doi:10.1371/journal.pmed.1003826)
Supplement: S1 Fig — (DOCX) [file pmed.1003826.s001.docx]

**S1 Fig: MMAT appraisals**

| **Authors** | **Study Design** | **Qualitative** | | | | | **Quantitative Descriptive** | | | | |
| --- | --- | --- | --- | --- | --- | --- | --- | --- | --- | --- | --- |
|  |  | **Is the qualitative approach appropriate to answer the research question?** | **Are the qualitative data collection methods adequate to address the research question?** | **Are the findings adequately derived from the data?** | **Is the interpretation of results sufficiently substantiated by data?** | **Is there coherence between qualitative data sources, collection, analysis and interpret- ation?** | **Is the sampling strategy relevant to address the research question?** | **Is the sample represent- ative of the target population?** | **Are the measurements appropriate?** | **Is the risk of nonresponse bias low?** | **Is the statistical analysis appropriate to answer the research question?** |
| Abel | Survey | - | - | - | - | - |  |  |  |  |  |
| Albright | Focus groups |  |  |  |  |  | - | - | - | - | - |
| Anonymous (CDCC) | Telephone Survey | - | - | - | - | - |  |  |  |  |  |
| Armstrong | Telephone Survey | - | - | - | - | - |  |  |  |  |  |
| Cameron | Focus groups |  |  |  |  |  | - | - | - | - | - |
| Casarin | Semi-structured interviews |  |  |  |  |  |  |  |  |  |  |
| Chen | Telephone Survey | - | - | - | - | - |  |  |  |  |  |
| Harris | Semi-structured interviews |  |  |  |  |  | - | - | - | - | - |
| Hebert | Survey | - | - | - | - | - |  |  |  |  |  |
| Kajikawa | Questionnaire |  |  |  |  |  |  |  |  |  |  |
| Kwong | Questionnaire | - | - | - | - | - |  |  |  |  |  |
| Kwong | Focus groups |  |  |  |  |  | - | - | - | - | - |
| Kwong | Survey | - | - | - | - | - |  |  |  |  |  |
| Lasser | Survey, observation |  |  |  |  |  |  |  |  |  |  |
| Lau | Telephone Survey | - | - | - | - | - |  |  |  |  |  |
| Nowalk | Telephone Survey | - | - | - | - | - |  |  |  |  |  |
| Phippards | Survey | - | - | - | - | - |  |  |  |  |  |
| Ramnadan | Online survey | - | - | - | - | - |  |  |  |  |  |
| Rikin | Survey | - | - | - | - | - |  |  |  |  |  |
| Schwartz | Survey | - | - | - | - | - |  |  |  |  |  |
| Sengupta | Interviews |  |  |  |  |  | - | - | - | - | - |
| Singleton | Survey | - | - | - | - | - |  |  |  |  |  |
| Sun | Survey, focus groups |  |  |  |  |  |  |  |  |  |  |
| Takahashi | Case-control | - | - | - | - | - |  |  |  |  |  |
| Winston | Telephone survey | - | - | - | - | - |  |  |  |  |  |
| Wooten | Telephone survey | - | - | - | - | - |  |  |  |  |  |
| Yu | Questionnaire | - | - | - | - | - |  |  |  |  |  |
| Yuen-Mai Siu | Interviews |  |  |  |  |  | - | - | - | - | - |
