# Supplementary material for: Vaccination uptake amongst older adults from minority ethnic backgrounds: A systematic review
Source: PLoS Med. 2021 Nov 4;18(11):e1003826. doi: 10.1371/journal.pmed.1003826 (PMC8568150; doi:10.1371/journal.pmed.1003826)
Supplement: S1 Table — (DOCX) [file pmed.1003826.s002.docx]

**S1 Table: GRADE CER-qual assessment***

| GRADE-CERqual assessments | | | | | |
| --- | --- | --- | --- | --- | --- |
| **No of studies** | **Methodological limitations component** | **Relevance component** | **Coherance component (An assessment of how clear and cogent the fit is between the data from the primary studies and the review finding)** | **Adequacy component (The degree of richness and quantity of data supporting a review finding)** | **Overall judgement** |
|  |  |  |  |  |  |
| Misinformation and lack of information | | | | | |
| 8 | Moderate confidence (3 studies rated low ROB; 5 studies rated moderate ROB) | Moderate confidence that studies are applicable to the context specified in the review question (population, intervention, outcome). Downgraded for partial relevance (Kwong et al) - majority Chinese population | High confidence across studies; little transformation of data/contradictory or ambiguous data | High confidence across studies; data saturation achieved | Moderate-High confidence |
| Perception of good health | | | | | |
| 7 | Moderate confidence (4 studies rated low ROB; 3 studies rated moderate ROB) | Moderate confidence that studies are applicable to the context specified in the review question (population, intervention, outcome). Downgraded for partial relevance (Kwong et al, Sun et al, Siu et al) - majority Chinese population | High confidence across studies; little transformation of data/contradictory or ambiguous data | High confidence across studies. Similar theme emerging across large number of participants. | Moderate-High confidence |
| Perception vaccine is ineffective | | | | | |
| 6 | Moderate confidence (1 study rated low ROB; 5 studies rated moderate ROB) | Moderate confidence that studies are applicable to the context specified in the review question (population, intervention, outcome). Downgraded for partial relevance (Kwong et al, Sun et al, Siu et al) - majority Chinese population | Moderate confidence across studies; some contradictory data on effectiveness of vaccination | Moderate confidence across studies | Moderate confidence |
| Perception vaccine causes harm | | | | | |
| 16 | High confidence (majority of studies rated low ROB) | High confidence that studies are applicable to the context specifified in the review question (population, intervention, outcome) | High confidence across studies; little transformation of data/contradictory or ambiguous data | High confidence across studies; emergent theme in 16 studies | High confidence |
| Mistrust in healthcare system | | | | | |
| 7 | High confidence (all studies rated low ROB) | Moderate confidence that studies are applicable to the context specified in the review question (population, intervention, outcome). Downgraded for partial relevance (2 studies) - majority Chinese population | High confidence across studies; little transformation of data/contradictory or ambiguous data | High confidence across studies | High confidence |
| Access | | | | | |
| 5 | Moderate confidence (4 studies rated low ROB; 1 study rated moderate ROB) | Moderate confidence that studies are applicable to the context specified in the review question (population, intervention, outcome). Downgraded for partial relevance (1 study) - majority Chinese population | High confidence across studies; little transformation of data/contradictory or ambiguous data | Moderate confidence across studies | Moderate confidence |
| Recommendation from a trusted healthcare professional (HCP) | | | | | |
| 10 | Moderate confidence (majority rated moderate ROB) | Moderate confidence that studies are applicable to the context specified in the review question (population, intervention, outcome). Downgraded for partial relevance - majority Chinese/Japanese population studies | High confidence across studies; little transformation of data/contradictory or ambiguous data | High confidence across studies | Moderate-High confidence |
| Vaccination reminders | | | | | |
| 6 | Moderate confidence (4 studies rated moderate ROB; 1 studies rated low ROB) | Moderate confidence that studies are applicable to the context specified in the review question (population, intervention, outcome). Downgraded for partial relevance - 1 study Chinese population studies | High confidence across studies; little transformation of data/contradictory or ambiguous data | Moderate confidence across studies | Moderate confidence |
| Knowledge of vaccinations and mechanism of action | | | | | |
| 6 | High confidence (4 low ROB; 2 moderate ROB) | Moderate confidence that studies are applicable to the context specified in the review question (population, intervention, outcome). Downgraded for partial relevance - majority Chinese/Japanese population studies | Moderate confidence across studies; some ambiguous data on knowledge of vaccinations and link as facilitator | Moderate confidence across studies | Moderate confidence |
| Supportive community | | | | | |
| 10 | Moderate confidence (majority rated moderate ROB) | Moderate confidence that studies are applicable to the context specified in the review question (population, intervention, outcome). Downgraded for partial relevance - majority Chinese/Japanese population studies | High confidence across studies; little transformation of data/contradictory or ambiguous data | High confidence across studies; rich data | Moderate-High confidence |
| Fear of developing disease | | | | | |
| 6 | Moderate confidence (majority rated moderate ROB) | Moderate confidence that studies are applicable to the context specified in the review question (population, intervention, outcome). Downgraded for partial relevance - majority Chinese/Japanese population studies | Moderate confidence across studies; some contradictory data | Moderate confidence across studies | Moderate confidence |
| Recognition of age as risk-factor | | | | | |
| 6 | Moderate confidence (majority rated moderate ROB) | Moderate confidence that studies are applicable to the context specified in the review question (population, intervention, outcome). Downgraded for partial relevance - majority Chinese/Japanese population studies | Moderate confidence across studies; some ambiguous data | Moderate confidence across studies | Moderate confidence |
| Vaccination setting | | | | | |
| 3 | Moderate confidence (all studies rated moderate ROB) | Moderate confidence that studies are applicable to the context specified in the review question (population, intervention, outcome). Downgraded for partial relevance - 1 study, majority Chinese population studies | High confidence across studies; little transformation of data/contradictory or ambiguous data | Low confidence across studies; few studies covered theme and unlikely data saturation achieved on theme | Moderate confidence |

*GRADE Confidence in the Evidence from Reviews of Qualitative Research: https://www.cerqual.org/
