# Supplementary material for: Vaccination uptake amongst older adults from minority ethnic backgrounds: A systematic review
Source: PLoS Med. 2021 Nov 4;18(11):e1003826. doi: 10.1371/journal.pmed.1003826 (PMC8568150; doi:10.1371/journal.pmed.1003826)
Supplement: S1 Text — (DOCX) [file pmed.1003826.s004.docx]

**S1 Text: Search Strategy for MEDLINE**

Search Run on 15 July 2021 informed by [1-7].

Ovid MEDLINE(R) and Epub Ahead of Print, In-Process & Other Non-Indexed Citations, Daily and Versions(R) <1946 to July 15, 2021>

1 ethnicity.ti,ab. 69303

2 ethnic.ti,ab. 85174

3 Minority Groups/ 14370

4 Ethnic Groups/ 63042

5 Population Groups/ 4896

6 continental population groups/ 22505

7 Hispanic Americans/ 28846

8 African Continental Ancestry Group/ 38162

9 American Native Continental Ancestry Group/ 473

10 asian continental ancestry group/ 67081

11 european continental ancestry group/ 67570

12 oceanic ancestry group/ 10570

13 African Americans/ 55398

14 arabs/ 4647

15 Asian Americans/ 7913

16 multi*cultural.ti,ab. 3018

17 multi cultural.ti,ab. 276

18 cross*cultural.ti,ab. 149

19 cross cultural.ti,ab. 12301

20 trans*cultural.ti,ab. 2262

21 transcultural.ti,ab. 2262

22 bame.ti,ab. 208

23 minority.ti,ab. 63771

24 minorities.ti,ab. 12515

25 1 or 2 or 3 or 4 or 5 or 6 or 7 or 8 or 9 or 10 or 11 or 12 or 13 or 14 or 15 or 16 or 17 or 18 or 19 or 20 or 21 or 22 or 23 or 24 426636

26 Vaccination/ 83419

27 Vaccines/ 22057

28 Immunization/ 51170

29 vaccin*.ti,ab. 324502

30 immuniz*.ti,ab. 134384

31 immunis*.ti,ab. 12414

32 26 or 27 or 28 or 29 or 30 or 31 435717

33 view*.ti,ab. 486393

34 barrier*.ti,ab. 312068

35 block*.ti,ab. 802853

36 obstacle*.ti,ab. 50720

37 hinder*.ti,ab. 61717

38 constrain*.ti,ab. 147222

39 facilitat*.ti,ab. 560165

40 attitud*.ti,ab. 158161

41 opinion*.ti,ab. 108567

42 belief*.ti,ab. 86124

43 perceiv*.ti,ab. 237173

44 perception*.ti,ab. 263701

45 aware*.ti,ab. 240710

46 personal view*.ti,ab. 2122

47 motiv*.ti,ab. 150884

48 reason*.ti,ab. 452246

49 incentiv*.ti,ab. 32512

50 limit*.ti,ab. 1744063

51 enabl*.ti,ab. 473939

52 influenc*.ti,ab. 1530868

53 factor*.ti,ab. 3496110

54 determinant*.ti,ab. 243268

55 challeng*.ti,ab. 883154

56 engage*.ti,ab. 169348

57 Attitude/ 48025

58 Motivation/ 68932

59 Patient Participation/ 26584

60 "Attitude to Health"/ 84233

61 Health Behavior/ 51412

62 33 or 34 or 35 or 36 or 37 or 38 or 39 or 40 or 41 or 42 or 43 or 44 or 45 or 46 or 47 or 48 or 49 or 50 or 51 or 52 or 53 or 54 or 55 or 56 or 57 or 58 or 59 or 60 or 61 9421072

63 older adult.ti,ab. 7785

64 older people.ti,ab. 29784

65 elder*.ti,ab. 265352

66 senior*.ti,ab. 42739

67 geriatri*.ti,ab. 49507

68 old age.ti,ab. 27351

69 late* life.ti,ab,kw. 18449

70 Aged/ 3149113

71 Geriatrics/ 30297

72 63 or 64 or 65 or 66 or 67 or 68 or 69 or 70 or 71 3315333

73 25 and 32 and 62 and 72 571

74 Infant/ 802657

75 Child/ 1715596

76 infant*.ti,ab. 414522

77 child*.ti,ab. 1428665

78 74 or 75 or 76 or 77 2737979

79 73 not 78 407

**References**

1. Sze S, Pan D, Nevill CR, Gray LJ, Martin CA, Nazareth J, Minhas JS, Divall P, Khunti K, Abrams KR, Nellums LB. Ethnicity and clinical outcomes in COVID-19: a systematic Review and Meta-analysis. EClinicalMedicine. 2020 Nov 12:100630.
2. Flu vaccination: increasing uptake - Evidence reviews for increasing uptake in health and social care staff. NICE guideline NG103. 2018. [online] NICE. Available at: (https://www.nice.org.uk/guidance/ng103/evidence/4-increasing-flu-vaccination-uptake-in-health-and-social-care-staff-pdf-6532083617) [Accessed 26 February 2021].
3. de Vries SG, Cremers AL, Heuvelings CC, Greve PF, Visser BJ, Bélard S, Janssen S, Spijker R, Shaw B, Hill RA, Zumla A. Barriers and facilitators to the uptake of tuberculosis diagnostic and treatment services by hard-to-reach populations in countries of low and medium tuberculosis incidence: a systematic review of qualitative literature. The Lancet Infectious Diseases. 2017 May 1;17(5):e128-43.
4. de Waard AK, Wändell PE, Holzmann MJ, Korevaar JC, Hollander M, Gornitzki C, de Wit NJ, Schellevis FG, Lionis C, Søndergaard J, Seifert B. Barriers and facilitators to participation in a health check for cardiometabolic diseases in primary care: A systematic review. European journal of preventive cardiology. 2018 Aug;25(12):1326-40.
5. Kanavaki AM, Rushton A, Efstathiou N, Alrushud A, Klocke R, Abhishek A, Duda JL. Barriers and facilitators of physical activity in knee and hip osteoarthritis: a systematic review of qualitative evidence. BMJ open. 2017 Dec 1;7(12).
6. Nair P, Bhanu C, Frost R, Buszewicz M, Walters KR. A systematic review of older adults’ attitudes towards depression and its treatment. The Gerontologist. 2020 Jan 24;60(1):e93-104.
7. Chaudhry UA, Wahlich C, Fortescue R, Cook DG, Knightly R, Harris T. The effects of step-count monitoring interventions on physical activity: systematic review and meta-analysis of community-based randomised controlled trials in adults. International Journal of Behavioral Nutrition and Physical Activity. 2020 Dec;17(1):1-6.
